# Analysis of the Emergent Climate Change Mitigation Technologies

**DOI:** 10.3390/ijerph18136767

**Published:** 2021-06-24

**Authors:** Deborah Panepinto, Vincenzo A. Riggio, Mariachiara Zanetti

**Affiliations:** Department of Engineering for Environment, Land and Infrastructures (DIATI), Politecnico di Torino, 10129 Torino, Italy; vincenzo.riggio@polito.it (V.A.R.); mariachiara.zanetti@polito.it (M.Z.)

**Keywords:** climate change mitigation, environmental impact assessment, negative emissions technologies, microalgae

## Abstract

A climate change mitigation refers to efforts to reduce or prevent emission of greenhouse gases. Mitigation can mean using new technologies and renewable energies, making older equipment more energy efficient, or changing management practices or consumer behavior. The mitigation technologies are able to reduce or absorb the greenhouse gases (GHG) and, in particular, the CO_2_ present in the atmosphere. The CO_2_ is a persistent atmospheric gas. It seems increasingly likely that concentrations of CO_2_ and other greenhouse gases in the atmosphere will overshoot the 450 ppm CO_2_ target, widely seen as the upper limit of concentrations consistent with limiting the increase in global mean temperature from pre-industrial levels to around 2 °C. In order to stay well below to the 2 °C temperature thus compared to the pre-industrial level as required to the Paris Agreement it is necessary that in the future we will obtain a low (or better zero) emissions and it is also necessary that we will absorb a quantity of CO_2_ from the atmosphere, by 2070, equal to 10 Gt/y. In order to obtain this last point, so in order to absorb an amount of CO_2_ equal to about 10 Gt/y, it is necessary the implementation of the negative emission technologies. The negative emission technologies are technologies able to absorb the CO_2_ from the atmosphere. The aim of this work is to perform a detailed overview of the main mitigation technologies possibilities currently developed and, in particular, an analysis of an emergent negative emission technology: the microalgae massive cultivation for CO_2_ biofixation.

## 1. Introduction and State of the Art

Climatology with the terms climate change or climate change refers to changes in the Earth’s climate, i.e., variations at different spatial scales (regional, continental, hemispherical, and global) and historical-temporal (decade, secular, millennial, and over-millennial) of one or more environmental and climatic parameters in their average values: temperatures (average, maximum, and minimum), precipitation, cloudiness, ocean temperatures, distribution, and development of plants and animals. Climate change is caused for the most part by greenhouse gas emissions.

Some gases present in the atmosphere, called greenhouse gases, are responsible for the greenhouse effect, which plays a fundamental role in the growth and development of life forms. Without serra gases, the earth would be frozen and lifeless. These Serra gases, although present in small quantities, favor the reflection towards the ground of IR rays (which determine the heating of surfaces) coming from the sun. The heat, therefore, remains stored in the atmosphere, resulting in a warming of the air and climate in a ratio “directly proportional” to the presence of Greenhouse gases. GHG can be of both natural and anthropic origin. The main greenhouse gases can have both origins: water vapor, carbon dioxide, nitrogen dioxide, and methane. There is also a wide range of greenhouse gases exclusively produced by human activity, such as alocarbons, the best known of which are chlorofluorocarbons, the emissions of which are regulated by the Montreal Protocol. [1,2]. Yue and Gao statistically analyzed global greenhouse gas emissions from natural systems and anthropogenic activities and concluded that the Earth’s natural system can be considered as self-balancing and that anthropogenic emissions add extra pressure to the Earth system [1].

In recent years, there has been a continuous energy consumption increase due to various activities. Reducing and decarbonizing energy consumption, especially with actions aimed at the most energy sectors (industry, buildings, and mobility) is, therefore, a way to go in order to reach “eco-sustainable” community [3]. Since 1870, more than 70% of anthropogenic greenhouse gas (GHG) emissions have resulted from the combustion of fossil fuels [4]. Constructing projections of future fossil fuel emissions for studies of future climate change is a challenging task. Workers in 19th-century mines could have scarcely imagined the technologies used by today’s coal industry. The same context is faced today when pondering an outlook for coal in the global energy system of the 21st-century [5]. Chakrabarty and Wang [6] highlighted that multinational enterprises must adapt their strategies to changes in the external business environment to perform environmentally, economically, and socially. This is because, on the one hand, their globalized activities across the world will have an important impact on the climate or on society [7]. Multinational enterprises can accelerate or slow sustainable development process both locally and globally by acting as one of the main actors in the international globalized economy [8,9,10]. Considering that cities are responsible for a large part of energy consumption (from which about 80% of carbon emissions derive) greening urban areas can also make a difference. In this case, it is necessary to intervene both in the reduction in emissions for energy consumption (due, for example, to domestic heating) and on emissions due to transport [11].

The decarbonization of development in the context of the Anthropocene was critically evaluated by Lugo–Morin D.R. [12] in a global scale. The study assess that the possibility of transitioning to a decarbonized global economy, or zero carbon emissions, is not encouraging indeed global energy production and carbon dioxide emissions are concentrated in a dozen countries and these nations are part of societies with an advanced social metabolism that negatively impacts the emission of CO_2_.

A new model for disaggregating the total observed changes into a number of source of change, thus opening to an estimated quantification of the effects originating from local policies alone was provided by Avezedo et al. [13]. Indeed, Avezedo et al. [13] remark that the evaluation of the effects of local actions for climate change mitigation is fundamental for the assessment of successful policies, but the existing methodologies do not bring to an single quantification of the effects of local policy actions. This happens because many causes of change in the local energy use and accountable emissions are not planned or controlled by the local authorities and national policies.

Considering the health risk point of view, Tong and Ebi [14] highlight that the global environmental changes are altering our planet in ways that be a model to current threats to human health, with the magnitude of these threats projected to grow over coming years if additional, proactive actions are not taken. The health risks of climate modification will become increasingly acute as climate change affects the quantity and quality of food and water, improves air pollution, changes the distribution of pathogens and disease transmission dynamics, and make less eco-physical buffering against extraordinary weather and climate events. Health systems urgently need to be upgraded to effectively address these emerging issues. In their study [14], the authors provide a global view of the health consequences of climate change, and discusses how health risks can be resized and avoided using mitigation and adaptation pathways.

Amelung et al. [15] describe effects of this type of health information on stated readiness to choose mitigation actions, as well as on simulation-based carbon emission reductions in a pre-recorded experimental setup among 308 households in 4 mid-size case-study cities in 4 European high-income countries: France, Germany, Norway, and Sweden. For every mitigation action from the food, housing, and mobility sectors, half of the sample received the amount of CO_2_ equivalents (CO_2_-eq) saved and the financial costs or savings the corresponding action generated. The remaining half additionally obtained information on direct health co-benefits, if applicable. For households, obtaining information on direct health co-benefits, a higher mean willingness to adopt food and housing actions was found, and a higher proportion very willing to choose one or more mitigation actions; and an increased simulated reduction in overall carbon footprint: difference in percent reduction equals to −2.70%, overall and −4.45%, for food.

A heated debate is open about the relationship between primary pollutants (air pollution) and greenhouse gases (GHG): there seems to be a link between the two, but the scientific evidence is not yet sufficient to state this with certainty. It is certain that the problem of primary pollution (PM, NOx, SOx, etc.) must always rediscover a central role given the consequences associated with it. From the point of view of GHG modeling, which is used for the production of future scenarios, it should be highlighted that existing models are not always able to adequately represent them. One of the most consolidated data are that direct emissions of atmospheric pollutants, such as black carbon, or those of secondary origin, such as sulphates and ozone, influence the radiative balance and, therefore, the climate change. However, if reducing the emissions of black carbon and the concentration of ozone (through a decrease in its precursors both anthropogenic and natural) could lead to a decrease in global temperature, a reduction in others pollutants, such as sulfates, would not have the same effect: in fact, these have on the atmosphere a cooling effect due to their ability to reflect solar radiation. From the point of view of the influence on climate change, it should be stressed that the increase in the greenhouse gas concentrations modifies the radiative balance between the atmosphere and the Earth’s surface, leading to a change in environmental conditions, including the temperatures and the meteorological regime increase. These evidences can determine changes in the atmosphere chemical transformations and, therefore, in the chemical composition of the same atmosphere. In particular, an increase in the temperatures and irradiation conditions could increase the ozone and secondary pollutants concentrations. The most important GHG is carbon dioxide, which persists in the atmosphere for thousands of years. Other important GHGs are methane, nitrous oxide and fluorinated gases. The first legally binding global climate agreement adopted by 195 countries in Paris (COP 21) in December 2015 includes the goal of limiting global warming to a maximum of two degrees in the long term (Paris Agreement). This will not be easy to achieve unless there are major improvements, particularly in the field of energy efficiency, which we know to be one of the main causes of global warming (Bel and Joseph, in the press). The IPPC has also pointed out in its fifth assessment report that it is necessary to reduce the global GHG emissions by 40–70% from the 2010 level before 2050, and to reduce the global GHG emissions to the level of near zero by the end of the 21st century [2]. To achieve the objectives set by IPCC, the development and use of adequate climate change mitigation technologies will play a pivotal and indispensable role [15,16,17]. In order to obtain this result for the GHG (and, in particular, for the carbon dioxide) it is certainly necessary to study and analyze the so-called “negative emission technologies” (technologies that allow a carbon dioxide concentrations reduction). It is necessary to emphasize that, considering the strategies in order to limit the climate change and, at the same time, to improve the air quality, it is necessary to assess the impacts of both these phenomena and, therefore, seek to identify synergies (win–win) and avoiding solutions that improve one of the two phenomena and worsen the other one (win–lose) [18]. The aim of this work is the analysis of the climate change mitigation phenomena coupled with the “negative technologies” particularly focusing on microalgae biofixation.

Therefore, the methodological approach used in the drafting of this paper was as follows: after a careful analysis of the state of the art of the phenomenon and of the technology currently available (also highlighting the state of maturity of the same), the topic of micro algae biofixation as an emerging and promising technology in the field of climate change mitigation was examined.

## 2. Climate Change Mitigation

The main GHG are carbon dioxide, methane, nitrous oxide, and the fluorinated gases, such as hydrofluorocarbons, perfluorocarbons, and sulphur hexafluoride [19].

These are the gas more analyzed in the scientific literature and defined and treated in the Kyoto Protocol and in the Paris Agreement [19]. 

As reported in the scientific literature the GHG are emitted by many economic activities: in particular the power generation is responsible for about 26%; the industries sector is responsible for about 19%; the transports sector is responsible for about the 13%; and finally the deforestation and forest degradation sector are responsible for about the 17% [20,21].

In the year 2018, the total GHG emissions were equal to 55.3 GtCO_2_e. These data come from the report prepared in the year 2019 by the United Nations Environment Programme (UNEP) [19]. 

Overall, 37.5 GtCO_2_ of the total 55.3 GtCO_2_e are attributed to fossil CO_2_ emissions deriving in particular from energy and power generation and from the industrial sector [19]. 

It is possible to note an increase of 2% in the year 2018 in comparison to an annual increase of 1.5% relative to the last decade for both the global GHG emissions and for the fossil Carbon dioxide emissions. This last increase (the increase in the emissions of the fossil CO_2_) is due, in particular, to the higher energy demand. The land-use change emissions amounted to 3.5 GtCO_2_ in the year 2018. In this year (2018), the emissions due to the land-use change and to the fossil CO_2_ accounted for approximately 74% of the total global greenhouse gas emissions. 

A recent Intergovernmental Panel on Climate Change (IPCC) report reported that the anthropogenic activities have caused until now a global warming until the 1.0 °C. In the same report are reported that global warming is likely to reach 1.5 °C between 2030 and 2052 if the current emissions will be not cut in the next years [19].

The terms “adaptation” and “mitigation” are two important terms that are fundamental in the climate change debate.

Climate mitigation is any action taken to permanently eliminate or reduce the long–term risk and hazard of climate change to human life. The Intergovernmental Panel on Climate Change (IPCC) [22] defines mitigation as: “An anthropogenic intervention to reduce the sources or enhance the sinks of greenhouse gases” [22].

Climate adaptation refers to the ability of a system to adjust to climate change (including climate variability and extremes) to moderate potential damage [23]. The IPCC defines adaptation as the “adjustment in natural or human systems to a new or changing environment”.

Mitigation refers to actions or policies that both reduce emissions of greenhouse gases that can cause climate change, or that increase the climate system’s capacity to treat such gases directly from the atmosphere (e.g., reforestation). The main gases that actively contribute to climate change can be indicated as carbon dioxide (CO_2_), methane (CH_4_), and nitrous oxide (N_2_O). Some human activities, for example energy generation from burning fossil fuels and deforestation and agriculture, emit these gases and contribute to increase their concentrations in the atmosphere. The actual concentrations of these gases in the atmosphere have reached values never seen for some 800,000 years or more [24].

As energy has a fundamental importance in modern economies, CO_2_ emissions have continued to increase rapidly in accordance with economic activity and population, despite international efforts under the UN Framework Convention on Climate Change (UNFCCC) to put under control the amount of CO_2_ in the atmosphere. The presence of methane and N_2_O in the atmosphere are also increasing. It must be considered that there is also a wide range of other substances that are relevant greenhouse gases but are presently in the atmosphere at much lower concentrations [24].

## 3. Applied Technologies for Mitigation

In order to ensure proper protection of the environment, it is necessary to operate in two ways: by means of pollution prevention techniques and by means of control techniques. These two techniques can be operated separately but to obtain good results it is better if they are applied jointly [25,26,27].

Prevention technologies act upstream, that is, before the pollutant is formed and by means of appropriate measures aim at its non-formation [27]. Pollution control technologies (also called end of pipe technologies) act downstream of the process, that is, when the pollutant is now formed: these technologies provide specific techniques or processes for the removal of the pollutant generated [25]. 

Wang (2017) [18] propose to classify the environmental technologies into 5 types:

- Eco-efficiency technology, the aim of this technology is reducing pollution by reducing the amount of required power and material in input to the process maintaining the same level of production; The purpose can be achieved by installing more energy-efficient equipment, by modifying the process, etc. The main advantages are related to the environmental and economic benefits: in fact, thanks to the use of eco-efficient technologies, significant economic savings are possible;
- Low-carbon energy technology, the aim of these kind of technologies is to operate a transition to Low-carbon energy technology (biofuel, wind, solar, etc.) from the conventional energy sources (coal, oil, and so on). Using these technologies, it is expected to protect the climate thanks to the lowest carbon emission quotas. However, there are two main problems:
  1. Higher costs of procuring or generating low carbon energy than conventional energy;
  2. Integrating low-carbon energy into existing energy supply systems can disrupt the current operational process.
- Green design technology, the aim of these kind of technologies is to reduce the pollutant contents of the products modifying, in particular, the design of the products (generally using more environmentally sustainable materials). The polluting contents of the product are usually measured on the basis of the life cycle analysis. Looking at technology from the demand side, “green” products are likely to become more attractive to environmentally conscious consumers. By analyzing the technology from the side of the company, we have that the design for regeneration or circulation can improve the recovery value of the products. Therefore, these technologies can have advantages both from an environmental point of view and from an economic point of view;
- Pollution control technology, these kind of technologies (call also end of pipe) aims to eliminate the pollution at the end of the process with appropriate instruments. Pollution control technology usually involves burning, recycling, filtering, and catalyzing pollutants. Typical pollution control technologies include electro-filters, bag filters, scrubbers (dry and wet), use of activated carbon, SNCR, or SCR systems [25,26];
- Management system technology, these kind of technologies try to reduce pollutants upstream, before their formation [27] adapting the way operations are handled. This is generally achieved through monitoring, reporting of pollution events and through employee training programs to raise awareness of climate change issues.
- Fawzy et al. [19] state that there are three main ways to mitigate climate change:
- Use of de-carbonization technologies and techniques in order to reduce CO_2_ emissions. These include the use of renewable energy instead of fossil fuels, the use of nuclear energy, the storage and use of carbon capture. These are, in the first case, well-established technologies;
- Use of so-called negative emissions technologies. These are recent technologies, which have not yet been studied in detail. These technologies are able to absorb CO_2_ present in the atmosphere. These include bioenergy carbon capture and storage, biochar, enhanced weathering, direct air carbon capture and storage, ocean fertilization [28];
- Technologies based on the principle of altering the balance of terrestrial radiation through the management of solar and terrestrial radiation. Such techniques are deducted forced radiative geoengineering technologies, and the main objective is stabilization or temperature reduction. At present, radiative techniques of forced geoengineering are studied only from the point of view of scientific research and are not included in policy frameworks.

### 3.1. The Negative Emissions Technologies

As reported from the European Parliamentary Research Service (EPRS) [29] there are a lot of technologies able to remove the CO_2_ from the atmosphere. In order to compare them with the traditional carbon abatement technologies an important parameter to consider is the amount of energy needed. In this sense most of the negative emissions technologies (NET) required only a minor increase in the fraction of energy that must be dediacted to these kind of technologies. This is a very important point that must be taken into consideration.

The negative emissions technologies (NET) are able to absorb the CO_2_ at low concentration present into the atmosphere. In order to perform this process the NET using an “industrial” process, which, depending on the NET used, can have different characteristics. This kind of process happens naturally during the photosynthesis and during the growth of the biomasses [29]. 

The Intergovernmental Panel on Climate Change (IPPC) in most of its scenarios of stabilization involves the use of NETs.

It is important to note that the use of NETs must not replace the indications relating to the cutting of GHG emissions. Using NETs and cutting emissions must implemented together in order to achieve good results [29].

The main negative emissions technologies are reported in the following. The information reported was elaborated starting from the reports present in the scientific literature [24,29,30]:

- Forestation, afforestation and reforestation would involve planting forests on unused land;
- Biochar, biochar involves the production of enriched carbon material by the slow pyrolysis process;
- Soil Carbon Management, agricultural land management practices such as reduced tilling, cover crops and certain grazing practices increase organic carbon levels in soils;
- Ocean Fertilization, involves adding nutrients to the ocean in order to stimulate the growth of planktonic algae and other microscopic plants that take up CO_2_;
- Augmented Ocean Disposal (“ocean liming”), uses lime in oceans to trap CO_2_ in a stable, dissolved inorganic form;
- Enhanced Weathering and Mineral Carbonation, implies the application of finely ground silicate or carbonate minerals to seawater or soils;
- Bio-energy with Carbon Capture and Storage (BECCS), is the combination of two mitigation options: biomass combustion to generate energy and Carbon Capture and Storage (CCS). The BECCS process achieve negative emissions by storing the carbon dioxide resulting from the combustion of plants, which have previously removed CO_2_ from the air through photosynthesis;
- Direct Air Capture, refers to industrial methods for removing carbon dioxide from the air by putting the air in contact with a chemical sorbent that are able to absorb the carbon dioxide. An example of this are the “Artificial Trees” technology: this is a technology that mimics the processes by plant life to withdraw CO_2_ from the atmosphere;
- Lime–Soda Process, this process is similar to artificial trees, but uses a chemical scrubbing method to enhance CO_2_ capture;
- Carbon Storage and CO_2_ utilization, both BECCS and Direct Air Capture need carbon storage to achieve permanent removal of the carbon from the atmosphere. The most common method is geological storage in depleted oil and gas fields, coal beds, and saline aquifers; however total storage capacity is uncertain and requires further geological studies.

Table 1 reports the net estimate costs (expressed as €/tCO_2_ removed) for the negative emissions technologies above reported [20].

Analyzing Table 1 shows that all the negative technologies reported have quite high CO_2_ removal costs. Enhanced weathering and mineral carbonation technology is the most expensive (about $1000 per ton of CO_2_ removed) followed by Ocean Fertilization (about $500 per ton of CO_2_ removed). The remaining technologies have quite similar costs (of the order of 100–150 $ per ton of CO_2_ removed).

These technologies are a concept rather, however, given the role that these technologies can play in mitigating climate change (absorbing a share of CO_2_ now present in the atmosphere) it is necessary to be able to give the right importance to these technologies and the right study or development.

Negative emission technologies to become industrially mature will also have to be studied and tested more individually. To date, in fact, most of the studies proposed by the IPCC include the implementation of negative emission technologies together with conventional decarbonization technologies [28], this is to achieve the objectives set by the Paris Agreement. Actually, only two negative technologies have been included in the IPCC’s assessments: bioenergy carbon capture and storage and afforestation and afforestation [30].

### 3.2. Microalgae for BIO-Fixation

The use of microalgae to actively bio fix CO_2_ is an activity strictly related with the growing factor of these microorganisms. In the past, a lot of research works were published where the focus was to produce enough biomass to economically sustain the production of biodiesel. This scope in the last years was slowly changed to production of food, cosmetic, and high value compounds. However, it is a fact that microalgae to successfully perform photosynthesis needs CO_2_, in fact it was reported that microalgae cells contain about 50% carbon, in which 1.8 kg of carbon dioxide are fixed by producing 1 kg of microalgae biomass [31,32]. Using photosynthesis process the CO_2_ is fixed by microalgae cells to support their growth by using the carbon to produce carbohydrate and consequently, the carbohydrates are used to build proteins, nucleic acids, and lipids [33]. Because of their simple cell structure and fast growth rate, microalgae are expected to have a 10 to 50 times higher CO_2_ bio fixation efficiency than terrestrial plants [34,35]. This aspect related to the microalgae metabolism could be usefully considered for carbon capture mitigation when the microalgae were cultivated on a high efficiency system that is strongly integrated with an industrial plant. In this scenario, the CO_2_ is provided by an existing industrial stream, as well as for the nutrient elements. There has been increasing interests on the use of microalgae growing technology for both bio fixation of carbon dioxide from flue gases [36,37,38,39,40,41] and removal of nutrients from wastewater [40,41,42,43,44]. Another consideration that needs to be done is related with the kind of light energy provided to the microalgae growing system. If the light is coming from natural source, it has been proven that the theoretical maxima of solar energy conversion efficiencies in photosynthesis is around 10%–8% solar-to-biomass [32]. As confirmed by other experimental works the best-case scenario (lab and small scale green microalgal productivities) has achieved only about 40% (or less) of the theoretical maximum productivity. At the same time, the best case solar to biomass energy conversion efficiency obtained with green microalgae did not exceed the 3% value [45,46]. Several studies reported by Melis (2009) [32], highlight solar to biomass energy conversion efficiencies for Spirulina and switchgrass that are lower than 1%. Obviously, biomass productivity is much lower for traditional C3 crop [47] and wild land plants, where the solar to biomass conversion efficiency values are below 0.1%.

If the light is provided by an artificial source that can provide the right quantity and quality of photon flux the global efficiency of the system can increase. First of all an optimized light spectrum that is specifically designed for the selected microalgae strain can double the energy conversion efficiency bringing the value from 8–10% to 20–22%. Secondary, the energy source used to power the growing system must come from a national energy mix that does not have coal, or from a renewable one with a high-power density value, for instance last generation of PV panels, wind, or tidal turbine. Only in this way the CO_2_ balance of the microalgae growing system can be compared with one that use natural light source. Finally, the growing system must allow a CO_2_ fixation rate, or removal percentage, as near as possible to the growth rate of the strain or to the 100% of removal. To achieve this result the system must be closed, not in direct contact with the atmosphere, with a well-known gas mass coefficient and with a CO_2_ inlet and O_2_ outlet mechanism carefully designed. In this way, the dissolved carbon dioxide inside the growing medium can be efficiently bio fixed by the microalgae, and the release of oxygen will not become a limiting factor. Using a closed growing system can allow to achieve another interesting result that is related with the protection of environment. Indeed, a closed system can allow to avoid microalgae contamination on the external environment and using a managed system for the water treatment. In this way, it can be possible to completely reuse the amount of water needed to the process, limiting its consumption and drastically reducing make-up cost with a positive environmental footprint. There is any industrial standard related with this specific kind of technology, even the scientific literature presents several difficulties as authors release research that cannot be directly compared to each other and the few commercial-industrial plant do not release any technical specifications. Despite the low efficiency obtainable from system placed under natural light and the scarcity of information related to technology that uses artificial light, the bio fixation of CO_2_ with microalgae is still under study by different research groups as its potential for climate change mitigation is only just beginning. In the work of Lim et al. [48], this aspect was deeply investigated as the authors selected a higher variety of published results. Additionally, they proposed a direct comparison of several experiments where the considered parameters were grouped under the carbon content quantification method used, then the evaluate carbon dioxide fixation rate and, consequently, the CO_2_ fixation efficiency. The first parameter is directly related with the biomass productivity and the second is more related with the growing technology used. At the end, after a comparison of more than 150 experimental tests the authors concluded assessing those microalgae have great potential not only to reduce the CO_2_ levels in the atmosphere, but the obtained biomasses are also useful in different type of applications. At the same time, they confirmed our initial thesis in relation with the fact that CO_2_ fixation quantification methods have not been critically analyzed and explicitly discussed in the scientific literature. For example, Almomani et al. [49] tested a pilot scale plant for a period of 20 months, verifying the bio fixation capacity and the growth rate of two different type of strains. The experiment was conducted with the use of a specific designed photobioreactor, where it was possible to use flue gas and wastewater as a viable source of nutrients. The conclusion was positive, but to be sure about the technology application the authors themselves suggested to improve the study with a scale up of the system. Only in this way will it be possible to recover data that can be used for assessing a valid economic feasibility and to perform a Life Cycle Analysis. 

## 4. Conclusions

In this work, the analysis of the climate change mitigation phenomena was performed. In particular, a review of the main “negative emissions technologies” or the technologies able to absorb the CO_2_ present in the atmosphere was conducted. For these technologies, the advantages and disadvantages and the cost are analyzed and reported (based on the literature data). The results of this first part show that the costs of these technologies are still very high and much still needs to be done in the field of research to make them industrially competitive. However, it is clear that these technologies can play a key role in achieving the objectives of the Paris Agreement. New information are added concerning an emergent negative emissions technologies: the microalgae.

Regardless, concerning the use of microalgae for CO_2_ bio-fixing more work is required, as the bio fixation efficiency is strictly related with the microalgae growth rate and to obtain a valid technology many difficult aspects still need to be solved. In this way, it will be possible to reduce the energy demand and reduce investment and operating costs.

## Figures and Tables

**Table 1:** Cost indication of the different negative emissions technologies (data from [20]).

NET	Average Costs ($/tCO_2_)
Forestation	100
Biochar	135
Soil Carbon Management	100
Ocean Fertilization	500
Augmented Ocean Disposal	90
Enhanced weathering and mineral carbonation	1000
Bioenergy with Carbon Capture and Storage (BECCS)	111
Direct Air Capture	95
Lime–Soda process	155
